# Positive fluid balance is associated with earlier acute kidney injury in COVID-19 patients

**DOI:** 10.62838/jccm-2026-0006

**Published:** 2026-04-30

**Authors:** Rezwan Munshi, Sandra Gomez-Paz, Salman Bhutta, Joshua Fogel, James Pellegrini, Narois Nehru, Eric Lam, Sofia Rubinstein

**Affiliations:** Nassau University Medical Center, East Meadow, NY, USA; Brody School of Medicine, East Carolina University, Greenville, NC, USA; Northwell Health, New Hyde Park, NY, USA; Brooklyn College, Brooklyn, NY, USA; Rutgers New Jersey Medical School, Newark, NJ, USA

**Keywords:** acute kidney injury, fluid balance, COVID-19, mortality, length of stay

## Abstract

**Introduction:**

Managing fluid balance in COVID-19 patients can be challenging, particularly if acute kidney injury (AKI) develops.

**Aim of the study:**

We study the relationship between fluid net input and output (FNIO) in COVID-19 patients with development of AKI, time to development of AKI, in-hospital length of stay (LOS), and in-hospital mortality.

**Material and Methods:**

Retrospective study of 403 patients with COVID-19. Data for FNIO were from day 1 through day 10 or earlier if AKI occurred.

**Results:**

AKI occurred in 22.8%, in-hospital mortality occurred in 26.3%, mean days to AKI were 7.7 (SD=6.3), and mean LOS was 11.5 (SD=13.2) days. In the multivariate logistic regression analyses, increased FNIO mean was significantly associated with slightly increased odds for mortality (OR=1.001, 95% CI:1.0001, 1.0011, p=0.02) but was not significantly associated with AKI. In the multivariate linear regression analyses, increased FNIO mean was significantly associated with lesser days to AKI (B=−6.63*10^−5^, SE=<0.001, p=0.003) in the whole sample, greater days to AKI in the subset of those with ICU treatment (B=<0.001, SE=<0.001, p<0.001), while FNIO mean was not significantly associated with LOS.

**Conclusions:**

Positive fluid balance was associated with faster onset of AKI and increased mortality. Fluid administration in patients with COVID-19 should be guided by routinely measuring FNIO. A restrictive fluid management regimen rather than usual care should be practiced.

## Introduction

COVID-19 is an acute respiratory infection that involves many organ systems including the renal system [1]. Acute kidney injury (AKI) incidence in COVID-19 patients ranges from 16.8% to 46.0% [1] and has a higher incidence of 50.6% in COVID-19 patients admitted to the intensive care unit (ICU) [2]. The mortality rate of COVID-19 patients with AKI is higher compared to those patients with AKI without COVID-19 [3].

Many variables are associated with increased risk for AKI in COVID-19 patients. There are demographic variables of age, male sex, and black race/ethnicity [3] associated with increased risk. Comorbidities of increased body mass index (BMI) [3], hypertension, and chronic kidney disease (CKD) [4] are associated with increased risk. ICU level of care [4] and medications [5] (e.g., nephrotoxins [4], vasopressors [3], and loop diuretics [5], are also associated with increased risk.

COVID-19-induced AKI can involve the kidneys with direct and/or indirect pathways [6]. Direct kidney involvement can occur with acute tubular necrosis via the angiotensin-converting enzyme 2 (ACE2) pathway [6]. Indirect kidney involvement can occur with COVID-19 inducing sepsis and hypoxia [6]. In addition, interactions between organs such as the lung, heart, and kidney can be another potential cause of the COVID-19-induced AKI [6]. Also, the generation of microthrombi in COVID-19 patients may lead to acute ischemic injury [6]. These mechanisms of COVID-19-induced AKI require effective fluid therapy strategies. Hospitalized COVID-19 patients often have sepsis and/or acute respiratory distress syndrome (ARDS) where high positive end-expiratory pressure can also cause hemodynamic changes [3]. Fluid management is an important component for treatment of sepsis [7] and ARDS [8]. Studies in non-COVID ARDS patients showed that restricting fluid intake decreased mortality [8]. The mainstay of treatment in patients with sepsis in non-COVID-19 patients is fluid resuscitation [7]. However, fluid management in AKI can be challenging as hypovolemia and hypervolemia [9] can worsen AKI.

The association of fluid management on the development of AKI in COVID-19 is unknown. Fluid net input and output (FNIO) as a marker of fluid status can be important for determining use of different fluid management strategies in patients with COVID-19. We study FNIO in COVID-19 patients over the initial 10-day period after presentation. Our primary aim is to study the association of fluid management with development of AKI in COVID-19 patients. Our secondary aims are to study the association of fluid management with time to development of AKI, mortality, and length of stay in hospital (LOS) in COVID-19 patients.

## Material and methods

### Participants

This was a retrospective study of 403 patients with confirmed COVID-19 with and without AKI who were admitted to a safety-net hospital in suburban New York City from March 1, 2020, through May 15, 2020. All patients were 18 years of age or older. Exclusion criteria were end-stage renal disease (on hemodialysis), severe liver disease, congestive heart failure New York Heart Association Class 4, and AKI occurring on the same day as admission. Diagnosis of COVID-19 was confirmed by a positive reverse transcriptase polymerase chain reaction (RT-PCR) showing SARS CoV-2 RNA. AKI was defined according to the Kidney Disease Improving Global Outcomes (KDIGO) criteria of either an increase in serum creatinine greater than or equal to 0.3 mg/dL within 48 hours, an increase in serum creatinine greater than or equal to 1.5 times baseline which is known or presumed to have occurred within the prior 7 days, or a urine volume less than 0.5 ml/kg/h for 6 hours [4]. All patients had fluid monitored for 10 days or until development of AKI which may have occurred earlier than 10 days. Institutional Review Board approval was received. A waiver for informed consent was obtained due to the retrospective nature of the study.

### Variables

The main predictor variable was FNIO mean. All available data for FNIO from the initial 10-day period after presentation was included. As many patients did not have data from these 10 days, the net mean was calculated for the available total FNIO over the 10 days divided by the number of days with data. For example, if someone had FNIO data for 2 days, mean FNIO consisted of the sum of the 2 values of FNIO divided by 2. If someone had FNIO data for 10 days, mean FNIO consisted of the sum of the 10 values of FNIO divided by 2.

Demographic variables were age (years), sex (male/female), and race/ethnicity (white, black, Hispanic, and other). Insurance status had categories of private, uninsured/emergency Medicaid (i.e., Medicaid issued during this hospitalization), regular Medicaid, and Medicare. Comorbidities analyzed were BMI, CKD, and the Charlson Comorbidity Index (CCI). CCI was calculated (range: 0–37) using age, history of myocardial infarction, heart failure, peripheral vascular disease, cerebrovascular accident, dementia, chronic obstructive pulmonary disease, connective tissue disease, peptic ulcer disease, chronic liver disease, diabetes mellitus, renal disease, solid tumor, leukemia, lymphoma, and AIDS status [10].

Intensive care unit (ICU) level of care was defined as hospitalization in a critical care area. Mechanical ventilation was recorded. Treatment and management consisted of use of vasopressors (epinephrine, norepinephrine, phenylephrine, vasopressin), nephrotoxins (acyclovir, amikacin, amoxicillin, amphotericin B, ampicillin, high dose aspirin, aztreonam, cephalosporin, contrast IV, diclofenac, enalapril, ertapenem, esomeprazole, gentamicin, ibuprofen, imipenem, ketorolac, lisinopril, losartan, meropenem, nafcillin, naproxen, pantoprazole, ramipril, valsartan, vancomycin), and/or loop diuretics (intravenous or oral). The outcome variables were development of AKI in-hospital (no/yes), days to AKI development, in-hospital mortality (no/yes), and LOS (days).

### Statistical Analysis

Mean and standard deviation were used to describe the continuous variables. Frequency and percentage were used to describe the categorical variables. Days to AKI and LOS were logarithmic transformed due to presence of skewness. Little’s Missing Completely at Random (MCAR) test was conducted for the FNIO days measured to determine if data were MCAR or systematically missing not at random (MNAR). The Pearson correlation analysis was conducted. Multivariate logistic regression was conducted for the categorical outcome variables. Multivariate linear regression was conducted for the continuous outcome variables. IBM SPSS Statistics version 30 was used for all analyses (IBM Corporation, Armonk, New York, 2024). All p-values were two tailed with alpha level for significance at p<0.05.

## Results

Table 1 shows the sample characteristics. The mean value of the FNIO mean variable was 612.2 mL (SD=747.38). Mean age was above 58 years. More than one third were female and more than half were Hispanic race/ethnicity. Almost one third had regular Medicaid insurance. Mean BMI was slightly below 30 kg/m^2^. Almost one quarter had mechanical ventilation. Nephrotoxin use was the most common treatment at 89.1%. For the outcome variables, AKI occurred in more than one fifth, mortality occurred in more than one quarter, mean days to AKI were 7.7 (SD=6.27), and mean LOS was 11.5 days (SD=13.17).

**Table 1.** Sample Characteristics of 403 Hospitalized COVID-19 Patients

**Variables**	**M (SD) or n (%)**
Net input and output average [mean]	612.2 (747.38)
Age (years) [mean]	58.1 (16.54)
Sex (female)	159 (39.5)

Race/ethnicity	
White	85 (21.1)
Black	79 (19.6)
Hispanic	214 (53.1)
Other	25 (6.2)

Insurance	
Private	105 (26.1)
Uninsured/emergency Medicaid	105 (26.1)
Regular Medicaid	132 (32.8)
Medicare	61 (15.1)

Body mass index (kg/m^2^) [mean]	29.8 (7.11)
Charlson comorbidity index [mean]	2.2 (2.11)
Chronic kidney disease (yes)	7 (1.7)
Intensive care unit (yes)	190 (47.1)
Mechanical ventilation (yes)	97 (24.1)
Nephrotoxin (yes)	359 (89.1)
Vasopressor (yes)	56 (13.9)
Loop diuretic (yes)	63 (15.6)

Outcomes	
Acute kidney injury (yes)	92 (22.8)
Mortality (yes)	106 (26.3)
Days to acute kidney injury [mean]	7.7 (6.27)
Length of stay (days) [mean]	11.5 (13.17)

Note: M=mean, SD=standard deviation

We conducted Little’s MCAR test for the 10 days of FNIO. There were non-significant p-values for the whole sample (p=0.90), among those without AKI (p=0.054), among those with in-hospital AKI (p=0.30), among those alive (p=0.06), and among those with in-hospital mortality (p=0.92). These non-significant values indicate that the null hypothesis that data are missing completely at random was retained and that the data were MCAR and not that the data were missing systematically MNAR. Table 2 shows the missing data percentages for the FNIO data. Missing data percentages ranged from 18.2% at day 1 among those with no mortality to as high as 88.4% at day 10 among those with no AKI. Table 3 shows the percentages for number of days having FNIO performed. There were varying percentages for number of days with 19.9% having FNIO performed only on one day. Also, even when FNIO was performed on more than one day, there were people who did not have FNIO performed on consecutive days such as FNIO performed on day 1, day3, and day 4 but not on day 2 (data not shown). Specifically, in the early phase of treatment, there were only 41.2% (n=166) that had FNIO performed consecutively from day 1 through day 4.

**Table 2.** Fluid Net Input and Output Missing Data for Each Day While In-Hospital

**Variable**	**Whole Sample Frequency (%) (n=403)**	**No AKI Frequency (%) (n=311)**	**Yes AKI Frequency (%) (n=92)**	**No Mortality Frequency (%) (n=297)**	**Yes Mortality Frequency (%) (n=106)**
On day 1	84 (20.8)	65 (20.9)	19 (20.7)	54 (18.2)	30 (28.3)
On day 2	103 (25.6)	81 (26.0)	22 (23.9)	78 (26.3)	25 (23.6)
On day 3	156 (38.7)	123 (39.5)	33 (35.9)	119 (40.1)	37 (34.9)
On day 4	189 (46.9)	149 (47.9)	40 (43.5)	145 (48.8)	44 (41.5)
On day 5	239 (59.3)	194 (62.4)	45 (48.9)	183 (61.6)	56 (52.8)
On day 6	270 (67.0)	216 (69.5)	54 (58.7)	206 (69.4)	64 (60.4)
On day 7	295 (73.2)	238 (76.5)	57 (62.0)	223 (75.1)	72 (67.9)
On day 8	320 (79.4)	252 (81.0)	68 (73.9)	238 (80.1)	82 (77.4)
On day 9	335 (83.1)	263 (84.6)	72 (78.3)	247 (83.2)	88 (83.0)
On day 10	349 (86.6)	275 (88.4)	74 (80.4)	256 (86.2)	93 (87.7)

Note: AKI=acute kidney injury

**Table 3.** Fluid Net Input and Output Total Number of Days Performed While In-Hospital

**Variable**	**Frequency (%) (n=403)**
1 day	80 (19.9)
2 days	89 (22.1)
3 days	41 (10.2)
4 days	46 (11.4)
5 days	23 (5.7)
6 days	30 (7.4)
7 days	22 (5.5)
8 days	13 (3.2)
9 days	18 (4.5)
10 days	41 (10.2)

LOS had a positive Pearson correlation with number of days when FNIO was performed (r=0.56, p<0.001). In analyses comparing LOS as a categorical variable, there were significantly greater mean number of days when FNIO was performed with those who had a longer LOS as compared to a shorter LOS. This pattern occurred for shorter LOS measured as 10 or less days (M=3.1, SD=2.06) versus longer LOS of 11 or more days (M=6.3, SD=3.35) with p<0.001 and also for shorter LOS measured as 20 or less days (M=3.9, SD=2.81) versus longer LOS of 21 or more days (M=6.4, SD=3.30) with p<0.001.

Table 4 shows multivariate logistic regression analyses for AKI. In all analytical models, FNIO mean was not significantly associated with AKI. In the whole sample and among those in the subset with ICU treatment, mechanical ventilation was significantly associated with increased odds for AKI. In the whole sample, Black race/ethnicity was significantly associated with increased odds for AKI. In the subset of those without ICU treatment, increased CCI was significantly associated with increased odds for AKI.

**Table 4.** Multivariate Logistic Regression Analyses for Acute Kidney Injury

**Variables**	**Whole sample OR (95% CI) (n=403)**	**p-value**	**No ICU OR (95% CI) (n=213)**	**p-value**	**Yes ICU OR (95% CI) (n=190)**	**p-value**
Net input and output average	1.00 (1.00, 1.00)	0.76	1.00 (1.00, 1.00)	0.48	1.00 (1.00, 1.00)	1.00

Age (years)	0.99 (0.96, 1.02)	0.39	0.97 (0.92, 1.02)	0.22	1.01 (0.97, 1.04)	0.76

Sex (female)	0.89 (0.44, 1.79)	0.74	2.15 (0.63, 7.37)	0.22	0.56 (0.22, 1.42)	0.22

Race/ethnicity						
White	1.00		1.00		1.00	
Black	2.99 (1.05, 8.51)	0.04	9.98 (0.97, 102.57)	0.053	1.62 (0.41, 6.49)	0.50
Hispanic	1.75 (0.68, 4.54)	0.25	4.44 (0.39, 50.95)	0.23	1.59 (0.50, 5.01)	0.43
Other	1.65 (0.41, 6.67)	0.48	<0.001 (<0.001,---)	1.00	1.80 (0.33, 9.65)	0.50

Insurance						
Private	1.00		1.00		1.00	
Uninsured/emergency Medicaid	0.63 (0.25, 1.57	0.32	1.01 (0.21, 4.93	1.00	0.43 (0.13, 1.42)	0.16
Regular Medicaid	0.58 (0.25, 1.35)	0.20	0.56 (0.12, 2.60)	0.46	0.62(0.21, 1.85)	0.39
Medicare	0.45 (0.15, 1.36)	0.16	1.33 (0.16, 11.08)	0.79	0.39 (0.10, 1.54)	0.18

Body mass index (kg/m^2^)	1.00 (0.96, 1.05)	0.88	1.02 (0.94, 1.10)	0.71	1.00 (0.95, 1.06)	0.99

Charlson comorbidity index	1.17 (0.94, 1.45)	0.15	1.39 (1.02, 1.91)	0.04	0.97 (0.71, 1.32)	0.84

Chronic kidney disease (yes)	1.55 (0.10, 22.02)	0.75	<0.001 (<0.001,---)	1.00	5.24 (0.17, 161.05)	0.34

Intensive care unit (yes)	1.54 (0.61, 3.91)	0.36	---	---	---	---

Mechanical ventilation (yes)	28.43 (11.77, 68.68)	<0.001	---	---	25.27 (10.33, 61.83)	<0.001

Nephrotoxin (yes)	1.16 (0.33, 4.15)	0.81	1.00 (0.23, 4.32)	1.00	4.78 (0.33, 69.01)	0.25

Vasopressor (yes)	1.20 (0.16, 8.96)	0.86	---	---	1.37 (0.20, 9.51)	0.75

Loop diuretic (yes)	1.09 (0.48, 2.51)	0.84	<0.001 (<0.001,---)	1.00	1.53 (0.60, 3.91)	0.37

Note: OR=odds ratio, CI=confidence interval, ICU=intensive care unit. In the model of no ICU, there were no patients with mechanical ventilation or vasopressor. Nagelkerke R Square: whole sample=0.51, no ICU=0.20, yes ICU=0.51.

Table 5 shows multivariate logistic regression analyses for mortality. In the whole sample and among those in the subset with ICU treatment, FNIO mean and age were each very slightly significantly associated with increased odds for mortality. In the whole sample and among those in the subset with ICU treatment, mechanical ventilation and vasopressor were each significantly associated with increased odds for mortality. In the whole sample, ICU treatment was significantly associated with increased odds for mortality.

**Table 5.** Multivariate Logistic Regression Analyses for Mortality

**Variables**	**Whole sample OR (95% CI) (n=403)**	**p-value**	**No ICU OR (95% CI) (n=213)**	**p-value**	**Yes ICU OR (95% CI) (n=190)**	**p-value**
Net input and output average	1.001 (1.0001, 1.0011)	0.02	1.00 (1.00, 1.00)	0.79	1.001 (1.0002, 1.0013)	0.01

Age (years)	1.05 (1.02, 1.08)	0.001	1.07 (0.99, 1.15)	0.09	1.04 (1.01, 1.08)	0.01

Sex (female)	1.48 (0.66, 3.35)	0.34	1.14 (0.19, 6.92)	0.89	1.61 (0.62, 4.24)	0.33

Race/ethnicity						
White	1.00		1.00		1.00	
Black	0.61 (0.19, 1.92)	0.39	0.85 (0.13, 5.58)	0.86	0.44 (0.10, 1.84)	0.26
Hispanic	0.62 (0.22, 1.74)	0.36	0.22 (0.02, 3.19)	0.27	0.85 (0.25, 2.90)	0.79
Other	0.50 (0.10, 2.56)	0.40	<0.001 (<0.001,---)	1.00	0.85 (0.14, 5.32)	0.86

Insurance						
Private	1.00		1.00		1.00	
Uninsured/emergency Medicaid	0.80 (0.25, 2.62)	0.72	1.77*10^7^ (<0.001,---)	1.00	0.70 (0.19, 2.57)	0.59
Regular Medicaid	2.57 (0.90, 7.35)	0.08	4.50*10^7^ (<0.001,---)	1.00	2.88 (0.88, 9.38)	0.08
Medicare	3.00 (0.85, 10.60)	0.09	3.23*10^7^ (<0.001,---)	1.00	2.50 (0.60, 10.45)	0.21

Body mass index (kg/m^2^)	1.00 (0.95, 1.06)	0.95	0.80 (0.61, 1.04)	0.09	1.04 (0.98, 1.10)	0.23

Charlson comorbidity index	1.06 (0.84, 1.34)	0.62	0.86 (0.53, 1.42)	0.56	1.15 (0.87, 1.51)	0.34

Chronic kidney disease (yes)	1.30 (0.10, 17.93)	0.84	3.11 (<0.001,---)	1.00	1.41 (0.07, 27.23)	0.82

Intensive care unit (yes)	10.18 (3.67, 28.22)	<0.001	---	---	---	---

Mechanical ventilation (yes)	23.46 (9.24, 59.57)	<0.001	---	---	23.16 (8.91, 60.19)	<0.001

Nephrotoxin (yes)	0.49 (0.12, 1.92)	0.30	0.50 (0.05, 5.08)	0.56	0.64 (0.08, 4.80)	0.66

Vasopressor (yes)	17.58 (1.23, 250.55)	0.03	---	---	17.24 (1.21, 245.50)	0.04

Loop diuretic (yes)	0.60 (0.22, 1.63)	0.32	<0.001 (<0.001,---)	1.00	0.67 (0.24, 1.89)	0.45

Note: OR=odds ratio, CI=confidence interval, ICU=intensive care unit. In the model of no ICU, there were no patients with mechanical ventilation or vasopressor. In the model of no ICU, the Box-Tidwell test suggested that the assumption of linearity of the logit for the Charlson comorbidity index was not met. An analytical model excluding the Charlson comorbidity index as a predictor variable had the same significance pattern as the model including the Charlson comorbidity index as a predictor variable. Data shown in the table is for the analytical model including the Charlson comorbidity index. Nagelkerke R Square: whole sample=0.69, no ICU=0.41, yes ICU=0.59.

Table 6 shows multivariate linear regression analyses for days to AKI. In the whole sample, FNIO mean was significantly negatively associated with days to AKI. However, among those in the subset with ICU treatment, FNIO mean was significantly positively associated with days to AKI. Black race/ethnicity was significantly negatively associated with days to AKI in the whole sample and in the subset without ICU treatment while this negative association for Hispanic race/ethnicity only occurred in the subset without ICU treatment. In the whole sample and among those in the subset with ICU treatment, mechanical ventilation was significantly negatively associated with days to AKI. In the whole sample and among those in the subset without ICU treatment, nephrotoxin was significantly positively associated with days to AKI. In the whole sample, ICU treatment was significantly positively associated with days to AKI. In the subset without ICU treatment, loop diuretic was significantly positively associated with days to AKI.

**Table 6.** Multivariate Linear Regression Analyses for Days to Acute Kidney Injury

**Variables**	**Whole sample B (SE) (n=403)**	**p-value**	**No ICU B (SE) (n=213)**	**p-value**	**Yes ICU B (SE) (n=190)**	**p-value**
Net input and output average	−6.63*10^−5^ (<0.001)	0.003	1.52*10^−5^ (<0.001)	0.65	<0.001 (<0.001)	<0.001

Age (years)	0.001 (0.001)	0.33	−0.001 (0.002)	0.78	0.002 (0.002)	0.22

Sex (female)	0.01 (0.03)	0.89	−0.05 (0.04)	0.29	0.07 (0.06)	0.26

Race/ethnicity						
White	Reference		Reference		Reference	
Black	−0.12 (0.05)	0.02	−0.15 (0.06)	0.02	−0.10 (0.09)	0.25
Hispanic	−0.07 (0.05)	0.12	−0.13 (0.06)	0.03	−0.04 (0.07)	0.61
Other	−0.04 (0.08)	0.57	−0.12 (0.10)	0.23	0.01 (0.11)	0.96

Insurance						
Private	Reference		Reference		Reference	
Uninsured/emergency Medicaid	0.06 (0.05)	0.23	0.05 (0.06)	0.39	0.07 (0.08)	0.34
Regular Medicaid	0.03 (0.04)	0.51	0.06 (0.06)	0.27	−0.01 (0.07)	0.86
Medicare	−0.03 (0.06)	0.59	0.02 (0.08)	0.77	−0.10 (0.09)	0.23

Body mass index (kg/m^2^)	−0.001 (0.002)	0.73	−0.002 (0.003)	0.58	−0.001 (0.003)	0.82

Charlson comorbidity index	0.004 (0.01)	0.71	0.02 (0.01)	0.19	−0.001 (0.02)	0.95

Chronic kidney disease (yes)	−0.21 (0.13)	0.11	−0.07 (0.22)	0.74	−0.23 (0.18)	0.20

Intensive care unit (yes)	0.23 (0.04)	<0.001	---	---	---	---

Mechanical ventilation (yes)	−0.21 (0.05)	<0.001	---	---	−0.18 (0.05)	<0.001

Nephrotoxin (yes)	0.18 (0.05)	<0.001	0.20 (0.06)	<0.001	0.07 (0.14)	0.62

Vasopressor (yes)	−0.01 (0.12)	0.95	---	---	−0.01 (0.13)	0.91

Loop diuretic (yes)	0.07 (0.05)	0.14	0.17 (0.08)	0.03	0.01 (0.06)	0.92

Constant	0.56 (0.12)	<0.001	0.63 (0.15)	<0.001	0.84 (0.23)	<0.001

Note: B=unstandardized beta, SE=standard error, ICU=intensive care unit. In the model of no ICU, there were no patients with mechanical ventilation or vasopressor. Adjusted R Square: whole sample=0.15, no ICU=0.09, yes ICU=0.13.

Table 7 shows multivariate linear regression analyses for LOS. In all analytical models, FNIO mean was not significantly associated with LOS. In the whole sample and among those in the subset with ICU treatment, mechanical ventilation was significantly positively associated with LOS. In the whole sample and among those in the subset without ICU treatment, nephrotoxin and loop diuretic were each significantly positively associated with LOS. In the whole sample, ICU treatment was significantly positively associated with LOS. In the subset without ICU treatment, Hispanic race/ethnicity was significantly negatively associated with LOS. In the subset without ICU treatment, vasopressor was significantly negatively associated with LOS.

**Table 7.** Multivariate Linear Regression Analyses for Length of Stay in Hospital

**Variables**	**Whole sample B (SE) (n=403)**	**p-value**	**No ICU B (SE) (n=213)**	**p-value**	**Yes ICU B (SE) (n=190)**	**p-value**
Net input and output average	−1.90*10^−5^ (<0.001)	0.39	1.83*10^−5^ (<0.001)	0.57	−2.97*10^−5^ (<0.001)	0.33

Age (years)	−0.001 (0.001)	0.70	0.001 (0.002)	0.79	−0.001 (0.002)	0.66

Sex (female)	−0.04 (0.03)	0.31	−0.04 (0.04)	0.32	−0.02 (0.06)	0.80

Race/ethnicity						
White	Reference		Reference		Reference	
Black	−0.03 (0.05)	0.62	−0.09 (0.06)	0.12	0.05 (0.09)	0.58
Hispanic	−0.01 (0.05)	0.77	−0.12 (0.06)	0.04	0.09 (0.07)	0.24
Other	−0.02 (0.08)	0.85	−0.12 (0.10)	0.22	0.11 (0.12)	0.32

Insurance						
Private	Reference		Reference		Reference	
Uninsured/emergency Medicaid	0.07 (0.05)	0.11	0.05 (0.06)	0.35	0.09 (0.08)	0.26
Regular Medicaid	−0.02 (0.04)	0.72	0.05 (0.05)	0.40	−0.08 (0.07)	0.23
Medicare	−0.05 (0.06)	0.44	0.02 (0.08)	0.77	−0.11 (0.09)	0.22

Body mass index (kg/m^2^)	−0.001 (0.002)	0.73	−0.001 (0.003)	0.81	−0.01 (0.004)	0.07

Charlson comorbidity index	−0.004 (0.02)	0.10	0.02 (0.01)	0.12	−0.01 (0.02)	0.75

Chronic kidney disease (yes)	−0.09 (0.13)	0.50	−0.11 (0.22)	0.61	−0.03 (0.18)	0.87

Intensive care unit (yes)	0.27 (0.04)	<0.001	---	---	---	---

Mechanical ventilation (yes)	0.14 (0.05)	0.003	---	---	0.16 (0.05)	0.004

Nephrotoxin (yes)	0.18 (0.05)	<0.001	0.17 (0.05)	0.002	0.18 (0.14)	0.23

Vasopressor (yes)	−0.24 (0.12)	0.051	---	---	−0.27 (0.13)	0.04

Loop diuretic (yes)	0.10 (0.05)	0.03	0.17 (0.08)	0.03	0.09 (0.06)	0.13

Constant	0.71 (0.12)	<0.001	0.16 (0.08)	0.04	1.07 (0.23)	<0.001

Note: B=unstandardized beta, SE=standard error, ICU=intensive care unit. In the model of no ICU, there were no patients with mechanical ventilation or vasopressor. Adjusted R Square: whole sample=0.29, no ICU=0.07, yes ICU=0.11.

## Discussion

We found that FNIO mean was significantly positively associated with slightly increased mortality in the whole sample and in the subset of those with ICU treatment, negatively associated with days to AKI in the whole sample, and positively associated with days to AKI in the subset of those with ICU treatment. FNIO mean was not significantly associated with development of AKI or LOS. Black race/ethnicity was significantly positively associated with AKI in the whole sample and negatively associated with days to AKI in the whole sample and in the subset of those without ICU treatment. Hispanic race/ethnicity was significantly negatively associated with days to AKI and LOS in the subset of those without ICU treatment. Increasing age was significantly associated with increased odds for mortality in the whole sample and in the subset of those with ICU treatment. Comorbidities of CCI was significantly associated with increased odds for AKI in the subset of those without ICU treatment. ICU treatment was significantly positively associated with mortality, days to AKI, and LOS. Mechanical ventilation was significantly positively associated with AKI, mortality, and LOS and significantly negatively associated with days to AKI. Vasopressor use was significantly positively associated with mortality and significantly negatively associated with LOS only in the subset of those with ICU treatment. Nephrotoxins were significantly positively associated with days to AKI and LOS in the whole sample and among the subset of those without ICU treatment. Loop diuretics was significantly positively associated with days to AKI among the subset of those without ICU treatment and with LOS in the whole sample and among the subset of those without ICU treatment.

We found that FNIO mean was positively associated with slightly increased mortality in the whole sample and in the subset of those with ICU treatment. FNIO mean was not significantly associated with development of AKI or LOS. Increased fluid administration in non-COVID-19 ARDS patients is associated with increased mortality [11]. Positive fluid balance in hospitalized sepsis patients is also associated with increased mortality [12]. A large percentage of COVID-19 patients develop sepsis [13] and ARDS [14]. Although we did not measure ARDS or sepsis in our sample, we believe that the likely high percentage of ARDS and sepsis patients in our sample could explain the increased mortality. In addition, previous research in non-COVID-19 patients with AKI suggests that positive fluid balance is associated with increased mortality [15]. Our finding in COVID-19 patients is similar to this pattern. The slightly increased odds of mortality are small. In the typical clinical setting, FNIO mean may not be associated with mortality. However, small values can best be understood from a public health perspective when understanding many people with COVID-19 where some small number of these people will have an association of FNIO mean with increased odds for mortality. It seems counterintuitive that FNIO mean was not significantly associated with development of AKI or LOS. We suggest that the average value of the FNIO mean was 612.2 mL which is a value associated with euvolemia that would not be associated with development of AKI or LOS.

In our study, FNIO mean was negatively associated with days to AKI in the whole sample and positively associated with days to AKI in the subset of those with ICU treatment. Although research in COVID-19 patients describes that positive fluid balance is a risk factor for the development of AKI [16], we did not find literature of an association between positive fluid balance and faster onset of AKI. We propose that the reason for our finding among those hospitalized patients with COVID-19 is due to a combination of renal insults (e.g., mechanical ventilation, sepsis, nephrotoxins, vasopressor use) in addition to positive fluid balance leading to faster onset of AKI [17]. However, the opposite pattern of a positive association of FNIO mean with days to AKI in the subset of patients in ICU is likely related to critical illness with vasodilation where positive fluid balance might be beneficial to maintain tissue perfusion initially [18]. It is also possible that the likely greater degree of positive fluid balance in ICU COVID-19 patients is associated with a dilutional effect of creatinine and delayed recognition of AKI, as previously described in non-COVID 19 patients [19]. We did not find any association between FNIO mean and LOS. We suggest that the higher mortality associated with increased FNIO mean, which would typically be associated with a shorter LOS, was possibly negated by the fact that AKI is typically associated with prolonged LOS in both non COVID-19 [20] and COVID-19 patients [21].

Black race/ethnicity was significantly positively associated with AKI in the whole sample and negatively associated with days to AKI in the whole sample and in the subset of those without ICU treatment. Hispanic race/ethnicity was significantly negatively associated with days to AKI and LOS in the subset of those without ICU treatment. Blacks have a higher frequency of the Apolipoprotein L1 (APOL1) gene variant that places them at increased risk for development of AKI among those with COVID-19 [22]. Also, blacks have a high prevalence of hypertension [23] which is a risk factor for early onset of AKI in COVID-19 patients [24]. We suggest that the potential presence of APOL1 genetic susceptibility and/or hypertension among those of black race/ethnicity was associated with earlier onset of AKI. Hispanics in the US have shorter height than whites or blacks resulting in smaller intravascular volume [25]. We suggest that Hispanics were more likely to have fluid overload and therefore have faster onset of AKI. Our Hispanic population is also relatively young and therefore likely to be healthier which is a possible reason for the negative association with LOS.

Increased age is associated with increased mortality in COVID-19 patients [26] and our findings for the whole sample and in the subset of those with ICU treatment are similar to this pattern. We found that COVID-19 patients who required ICU level of care had an association with increased odds for mortality, longer days to AKI, and longer LOS but not for developing AKI. We found that COVID-19 patients with AKI that required ICU level of care was associated with longer days to AKI. Previous research found that sepsis is associated with delayed onset of AKI in non-COVID-19 patients admitted to the ICU [27]. One study showed that the later the onset of AKI was associated with more severe AKI [17] in critically-ill COVID-19 patients, similar to our findings. It is possible that the likely greater degree of positive fluid balance in ICU COVID-19 patients results in a dilutional effect of creatinine and delayed recognition of AKI, as previously described in non-COVID 19 patients [19]. It is also likely that delayed recognition of AKI and increased mortality in the ICU subset resulted in no association with the development of AKI. The positive correlation of ICU level of care with mortality and LOS in COVID-19 patients has been reported in previous studies [28], which is similar to our findings. We hypothesize that this is due to ICU patients having more severe illness which leads to a higher mortality rate and longer duration of hospitalization.

Mechanical ventilation was significantly positively associated with AKI, mortality, and LOS and significantly negatively associated with days to AKI. These findings are consistent with studies of COVID-19 patients requiring mechanical ventilation that they had increased risk of mortality [28], increased risk of AKI [3] and more severe AKI [29]. Mechanical ventilation requirement in COVID-19 patients in the ICU is also associated with an earlier onset of AKI [3,24] as is seen in our study. The increased LOS is also consistent with those showing that COVID-19 patients requiring mechanical ventilation are sicker and therefore require prolonged hospital stay [28].

Vasopressor use was significantly positively associated with mortality and significantly negatively associated with LOS only in the subset of those with ICU treatment. A study showed that patients that required vasopressors showed higher severity of AKI with increased mortality rates [29], which is similar to our mortality findings. We found a negative association between vasopressor use and LOS. There is high mortality among those with vasopressor use [28]. We suggest that in our study the mortality among those with vasopressor use was the factor for decreased LOS.

Nephrotoxins were significantly associated with increased days to AKI and increased LOS in the whole sample and among the subset of those without ICU treatment. Loop diuretics were significantly associated with days to AKI among the subset of those without ICU treatment and with increased LOS in the whole sample and among the subset of those without ICU treatment. There is a paucity of data regarding the association of time to development of AKI and nephrotoxin use in COVID-19 patients. One study reports that AKI developing three to five days after admission is associated with nephrotoxic exposure in patients with severe COVID-19 [17]. Our finding differs and may be due to lower nephrotoxin exposure earlier during hospitalization as management with antibiotics was initially relatively restricted given the viral nature of the disease. We suggest that the nephrotoxin burden was higher later in the course of the disease due to worsening clinical status and to the treatment concomitant sepsis and other co-infections, hence the longer time to onset of AKI in our patients. With regard to LOS, one study found that the ongoing exposure to nephrotoxins due to more complicated course of disease results in prolonged LOS among COVID-19 patients [30], which is consistent with our findings. Also, similar to our findings, nephrotoxin use is associated with increased LOS in pediatric COVID-19 patients with AKI [31]. Moreover, diuretic use is associated with AKI in critically-ill COVID-19 patients [32], who typically have longer LOS [32], which could explain our findings.

For comorbidities, history of CKD and BMI were not associated with any outcome. CCI was significantly associated with increased odds for AKI in the subset of those without ICU. There is a paucity of data regarding the timing of onset of AKI and its association with history of CKD in COVID-19 patients. We did not show that COVID-19 patients with underlying CKD have a faster onset of AKI or a greater risk of developing AKI. Previous research reports increased mortality in COVID-19 patients with severe obesity (BMI >40kg/m^2^) but no association with mortality in patients who are overweight (BMI 25–29.9 kg/m^2^) or with class 1 obesity (BMI 30–34.9 kg/m^2^) [33]. As our sample BMI mean was approximately 30 kg/m^2^, which is the defining point between overweight and obesity, it is possible that is why we did not find an association. While some studies report that increased CCI is associated with worse outcomes in COVID-19 patients overall [34], particularly with CCI >3 [35], others did not find an association in COVID-19 patients with AKI [36]. Our findings for ICU patients are consistent with those reporting no association. However, our study found that CCI was significantly associated with increased odds for AKI in the subset of those without ICU. This is consistent with findings of other studies of general hospitalized COVID-19 patients [37]. It is possible that as ICU patients have more important covariates such as mechanical ventilation that CCI was no longer significantly associated with AKI.

It is important to consider potential bias that can occur while conducting the study. We believe that there are no concerns of selection or sampling bias, as we used all patients with available data and believe our sample is representative of our population. We believe that there are no concerns of measurement bias, as our measurement approach of using the mean for whatever FNIO were available provides a useful measure for understanding FNIO. Lastly, patients with shorter hospital stays had less FNIO data. This is exactly what one would expect. If a patient has less days in hospital there would be less data for FNIO and any other variable that would be measured on multiple days. Our analysis of using the mean value for whatever FNIO data were available lessens any possible survivorship bias, as our FNIO data are not based upon number of days.

A study strength is that this appears to be the first COVID-19 study looking at the association of FNIO with development of AKI, days to AKI, mortality, and LOS. This study has some limitations. First, when baseline creatinine level was unknown, we used the lowest recorded creatinine level. Second, we excluded patients who did not have FNIO recorded. Third, our study was done at a single safety-net hospital and may not generalize to other healthcare settings. Fourth, our study was done in the early phase of the pandemic, during which standardized treatment guidelines were not available. As COVID-19 treatment protocols have developed since that time, our findings may not be applicable to later waves. Fifth, we did not have data for ARDS which could potentially contribute to the analytical models.

## Conclusion

We found that FNIO mean was positively associated with slightly increased mortality in the whole sample and in the subset of those with ICU treatment, negatively associated with days to AKI in the whole sample, and positively associated with days to AKI in the subset of those with ICU treatment. This sheds light on the importance of appropriate fluid management in COVID-19 patients, particularly in the critically-ill, and the association with kidney function. Physicians should be aware of this association and strive for euvolemia in managing fluid status in order to prevent AKI and subsequent poor outcomes with such COVID-19 patients. Fluid administration in patients with COVID-19 should be guided by routinely measuring FNIO. A restrictive fluid management regimen rather than usual care should be practiced.
